# Clinical and Functional Genetic Characterization of the Role of Cardiac Calcium Channel Variants in the Early Repolarization Syndrome

**DOI:** 10.3389/fcvm.2021.680819

**Published:** 2021-06-18

**Authors:** Xiu Chen, Hector Barajas-Martínez, Hao Xia, Zhonghe Zhang, Ganxiao Chen, Bo Yang, Hong Jiang, Charles Antzelevitch, Dan Hu

**Affiliations:** ^1^Department of Cardiology and Cardiovascular Research Institute, Renmin Hospital of Wuhan University, Wuhan, China; ^2^Hubei Key Laboratory of Cardiology, Wuhan, China; ^3^Lankenau Institute for Medical Research, Lankenau Heart Institute, Wynnewood, PA, United States; ^4^Sidney Kimmel Medical College, Thomas Jefferson University, Philadelphia, PA, United States

**Keywords:** early repolarization syndrome, calcium channel, gene mutation, trafficking, sudden cardiac death

## Abstract

**Background:** Early repolarization syndrome (ERS) is an inherited sudden cardiac death (SCD) syndrome. The present study investigates the role of genetic variants in cardiac calcium-channel genes in the pathogenesis of ERS and probes the underlying mechanisms.

**Methods:** Polymerase chain reaction–based next-generation sequencing was carried out using a targeted gene approach. Unrelated ERS probands carrying calcium-channel variants were evaluated clinically and compared with matched healthy controls. Wild-type (WT) and mutant *CACNA1C* genes were coexpressed with *CACNB2b* and *CACNA2D1* in HEK293 cells and studied using whole-cell patch-clamp techniques and confocal fluorescence microscope.

**Results:** Among 104 ERS probands, 16 carried pathogenic variants in calcium-channel genes (32.2 ± 14.6 years old, 87.5% male). The symptoms at diagnosis included syncope (56.3%), ventricular tachycardia/fibrillation (62.5%), and SCD (56.3%). Three cases (18.8%) had a family history of SCD or syncope. Eight patients (50.0%) had a single calcium gene rare variant. The other half carried rare variants in other ERS-susceptible genes. Compared with controls, the heart rate was slower (72.7 ± 8.9 vs. 65.6 ± 16.1 beats/min, ^*^p < 0.05), QTc interval was shorter (408.2 ± 21.4 vs. 386.8 ± 16.9 ms, ^**^p < 0.01), and Tp-e/QT was longer (0.22 ± 0.05 vs. 0.28 ± 0.04, ^***^p < 0.001) in single calcium mutation carriers. Electrophysiological analysis of one mutation, *CACNA1C*-P817S (c.2449C>T), revealed that the density of whole-cell calcium current (*I*_Ca_) was reduced by ~84.61% compared to WT (−3.17 ± 2.53 vs. −20.59 ± 3.60 pA/pF, *n* = 11 and 15, respectively, ^**^p < 0.01). Heterozygous expression of mutant channels was associated with a 51.35% reduction of *I*_Ca_. Steady-state inactivation was shifted to more negative potentials and significantly accelerated as well. Confocal microscopy revealed trafficking impairment of *CACNA1C*-P817S (peripheral/central intensity: 0.94 ± 0.10 in WT vs. 0.33 ± 0.12 in P817S, *n* = 10 and 9, respectively, ^**^p < 0.01).

**Conclusions:** ERS associated with loss-of-function (LOF) genetic defects in genes encoding the cardiac calcium channel represents a unique clinical entity characterized by decreased heart rate and QTc, as well as increased transmural dispersion of repolarization. In the case of *CACNA1C*-P817S, impaired trafficking of the channel to the membrane contributes to the LOF.

## Introduction

An early repolarizationpattern (ERP), defined as J-point elevation ≥1 mm in ≥2 contiguous inferior and/or lateral leads of a standard 12-lead electrocardiogram (ECG), was traditionally considered as a normal electrocardiographic variant with a benign outcome for several decades (1–4). However, in 2008, Haïssaguerre et al. demonstrated a definitive relationship between ERP and idiopathic ventricular fibrillation (IVF), supporting the hypothesis of Gussak and Antzelevitch in 2000 that ERP is not always benign and may be malignant in some cases (5, 6). Numerous clinical and experimental observations have since confirmed the association between ERP and fatal arrhythmias (7–9), giving rise to the term *early repolarization syndrome* (ERS). Thus, ERP in a patient resuscitated from otherwise unexplained ventricular fibrillation (VF)/polymorphic ventricular tachycardia (VT) is referred to as ERS (1).

ERS has a propensity for heritability (10, 11). Eight ion-channel genes (*KCNJ8, ABCC9, SCN5A, SCN10A, KCND3, CACNA1C, CACNB2b*, and *CACNA2D1*) have been associated with ERS in recent years (12–17). Brugada syndrome (BrS) patients carrying calcium-channel mutations have been reported to have briefer QTc intervals and greater risk for cardiac events and sudden cardiac death (SCD) (18). Only a small fraction of calcium mutations associated with ERS have been functionally analyzed to ascertain causality and establish a plausible contribution to pathogenesis. Thus, the contribution of cardiac calcium-channel mutations to the etiology of ERS remains unclear.

The l-type calcium channel (LTCC) is a multisubunit protein complex composed of four subunits: the main pore-forming α1 (Ca_V_1.2) subunit encoded by *CACNA1C*, which determines the main biophysical and pharmacologic properties of the channel, and three auxiliary subunits, including a cytoplasmic β subunit encoded by *CACNB*, α2δ subunit encoded by *CACNA2D*, and a γ subunit encoded by *CACNG* (19–21).

We sought to identify genetic variations in the α1, β2, and α2δ1 subunits of LTCC among probands diagnosed with ERS and to investigate the potential underlying mechanism. Clinical characterization and functional genomic identification are investigated in detail.

## Methods

### Clinical Analysis

This study was approved by Renmin Hospital of Wuhan University Institutional Review Board and performed in accordance with the Declaration of Helsinki. The study population consisted of 104 cases diagnosed with ERS and 150 healthy controls with no family history of cardiac arrhythmias. All participants underwent clinical and genetic studies after obtaining informed consent. Patients were diagnosed with ERS based on established criteria (1, 2). Gender, age at diagnosis, clinical presentation, family history of SCD, and results of genetic screening were assessed. ECG parameters, including P wave duration, PR interval, QT interval, rate corrected QT interval, and QRS duration, were measured from lead II of 12-lead ECGs. ERP in the lateral leads is referred to as ERS type 1, in inferolateral leads is type 2, and with a global pattern (inferolateral + anterior or right ventricular leads) is type 3.

### Mutation Analysis

Genomic DNA was extracted from peripheral blood leukocytes of patients with a standard protocol and amplified by polymerase chain reaction (PCR) on GeneAmp PCR System 9700 (Applied Biosystems, Foster City, CA, USA). A panel designed using the tool Array (Agilent Technologies, Inc.) was used to directly sequence targeted genes previously associated with cardiac arrhythmias, including ERS. PCR products were purified with reagent (ExoSAPIT, USB, Cleveland, OH, USA) and directly sequenced from both directions using an ABI PRISM 3730 Automatic DNA Analyzer (Applied Biosystems). The sequencing results were analyzed by Mutation Survey V4.0.8 software (Softgenetics, USA) and reconfirmed by the above procedures. Novel variants considered to be pathogenic were either (1) stop/frameshift variants; (2) missense mutations located in the amino acid conservative region across species; (3) splice-site variations meeting the GT-AT rules; (4) minimum allele frequency (MAF) in the control population ≤0.003; or (5) predicted to be “possibly damaging” or “disease-causing” by the bioinformatics programs of SHIFT, PolyPhen-2, PROVEAN (Protein Variation Effect Analyzer) and MutationTaster2.

### Site-Directed Mutagenesis and Transfection of the HEK293 Cell

For the patch-clamp study, site-directed mutagenesis was performed on full-length human wild-type (WT) and mutant *CACNA1C* cDNA cloned in pCDNA3.1 vector tagged with enhanced yellow fluorescent protein (EYFP). cDNA of WT-*CACNB2b* and WT-*CACNA2D1* genes both cloned in pcDNA3.1 vector. Mutated genes were functionally expressed in human embryonic kidney (HEK293) cells as previously described (17). cDNAs of the three LTCC subunits were cotransfected with a 1:1:1 molar ratio using Lipofectamine 2000 reagent (Invitrogen™, Carlsbad, CA, USA). Electrophysiological studies were performed after 48 to 72 h of incubation.

### Electrophysiology Study

Calcium currents were recorded in HEK293 cells using whole-cell, patch-clamp techniques at room temperature (20–24°C) with Axon-700B patch-clamp amplifiers and pCLAMP10.4 software (Axon Instruments, Sunnyvale, CA, USA). HEK293 cells were placed in the experimental groove over an inverted microscope (IX70; Olympus, Tokyo, Japan) and perfused with a corresponding external solution containing the following (in mmol/L): glucose 10, CaCl_2_ 2, MgCl_2_ 1, HEPES 10, and TEA 150, pH 7.35 with CsOH). Patch pipettes, made from 1.5-mm OD borosilicate glass capillaries, were filled in a solution containing the following (in mmol/L): CsCl 110, CaCl_2_ 0.1, HEPES 10, EGTA 10, MgATP 2, and TEA 10 (pH 7.3 with CsOH), with uncompensated access resistances of 1.5 ± 0.7 MΩ. All currents were filtered at 1 kHz and digitized at 5 kHz with an eight-pole Bessel filter. Series resistance was electronically compensated at 70–80%.

Whole-cell calcium current (*I*_Ca_) was constructed with voltage steps by applying 400-ms pulses from a holding potential of −90 mV, to potentials ranging between −60 and +60 mV, in 10-mV steps. Voltage dependence of the steady-state voltage-dependent inactivation of *I*_Ca_ was evoked by a dual-pulse protocol with a 400-ms conditioning pulse from −90 mV to potentials between −100 and +20 mV. A Boltzmann function was fitted to the conductance-voltage and inactivation or activation curves, yielding the midpoint (*V*_½_) and slope (*k*) value of the curves.

### Localization of Ca^2+^ Channels

Channel trafficking was assessed using Ca^2+^ channels tagged with EYFP by confocal microscopy, as previously described (18). Cells were experimented 48 h after transfection by a Leica confocal laser scanning microscopy (Leica Microsystems, Heidelberg, Germany). EYFP-labeled cells were analyzed in the XYZ configuration. A region of interest was restricted within 2 μm of the plasma membrane, and the average pixel intensity within this region was referred to as peripheral staining, with average pixel intensity for the remaining part of the cell defined as central staining. The ratio of peripheral to central intensity was calculated.

### Statistical Analysis

Statistical analysis was carried out using GraphPad software version 8.0. A normality test was performed to assess the distribution of the data before applying a parametric test. Continuous variables were expressed as the mean ± standard error and evaluated using the Student *t*-test between two groups and one-way analysis of variance between multiple groups. Descriptive statistics for categorical variables were presented as count and percentages. Statistical significance was defined as *p* < 0.05.

## Results

### Clinical Characterization

Among the 104 unrelated patients diagnosed with ERS who underwent genetic screening for ion-channel gene mutations, 16 (15.4%) were found to carry a variant in one of the calcium-channel genes. The clinical characteristics of patients displaying a calcium-channel gene variant were summarized in Table 1. The average age at diagnosis of ERS was 32.2 ± 14.6 years; 87.5% were males. The symptoms at diagnosis included syncope (56.3%), VT/VF (62.5%), SCD (56.3%), and other atypical symptoms (37.5%); none were asymptomatic. Three cases (18.8%) had a family history of SCD or syncope. Three presented with atrial fibrillation (AF), five (31.3%) with cardiac conduction disease (CCD), and four (25.0%) with bradycardia. Of the 16 ERS patients carrying calcium-channel variant(s), 1 displayed J-point elevation localized to the lateral leads (ERS1), 5 localized to inferior leads (ERS2), and 10 presented with a global pattern (ERS3).

**Table 1:** Clinical characteristics of ERS probands carried calcium mutation.

**Index**	**ERS cases**
Age (years)	32.2 ± 14.6
**Gender**	
Male, *n* (%)	14 (87.5)
Female, *n* (%)	2 (12.5)
**Symptom**, ***n*** **(%)**	
Syncope	9 (56.3)
SCD/ASCD	9 (56.3)
VT/VF	10 (62.5)
Atypical symptom	6 (37.5)
Asymptomatic	0
AF, *n* (%)	3 (18.8)
Bradycardia, *n* (%)	8 (50.0)
CCD, *n* (%)	5 (31.3)
Family history of SCD/syncope, *n* (%)	3 (18.8)
**Type**, ***n*** **(%)**	
ERS1	1 (6.3)
ERS2	5 (31.3)
ERS3	10 (62.5)
**Probands with calcium mutations**	
Single calcium mutation^*^	8 (50.0)
With additional mutation(s)^#^	8 (50.0)

* *Variants from CACNA1C, CACNB2b, CACNA2D1*.

# *Combined with extra mutations in SCN5A, SCN10A, ABCC9, KCNJ11, etc*.

**Figure 1:**
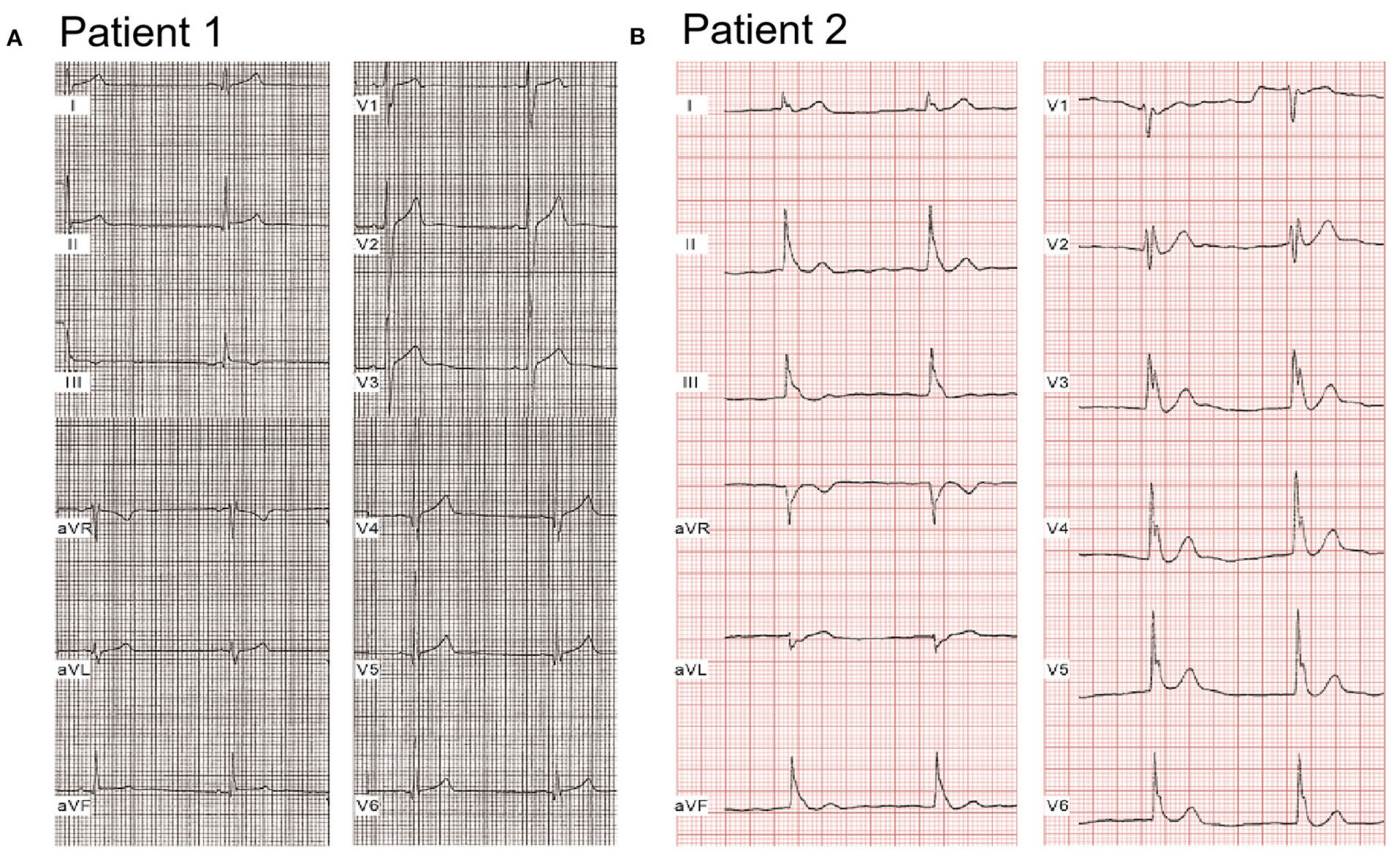
Representative electrocardiograms of two ERS probands with *CACNA1C*. **(A)** Patient 1 was a 20-year-old male patient, identified with a mutation, P817S, in *CACNA1C*. He suffered syncopal episodes twice while exercising. ECGs showed bradycardia (HR = 46 bpm) and an ERP with notching in the inferior leads, leading to a diagnosis of ERS type 2. **(B)** Patient 2, a 17-year-old young male patient, presented with spontaneous VF on the background of syncope. ECGs exhibited sinus bradycardia (HR = 50 bpm) and an ERP with notching/slur in a global pattern, thus diagnosed with ERS type 3.

Compared with healthy controls, heart rate was significantly slower [72.7 ± 8.9 beats/min (bpm), 65.6 ± 16.1 bpm, 60.9 ± 9.6 bpm; *p* < 0.05, *p* < 0.001 vs. controls, respectively]; QTc interval was significantly shorter (408.2 ± 21.4 ms, 386.8 ± 16.9 ms, 389.5 ± 23.6 ms; *p* < 0.01, *p* < 0.05 vs. controls, respectively), and Tp-e/QT was significantly longer (0.22 ± 0.05 vs. 0.28 ± 0.04 vs. 0.26 ± 0.07; *p* < 0.01, *p* < 0.05 vs. controls, respectively) in both single calcium mutation carriers and cases with additional mutation(s) (Table 2). Figure 1 shows a representative 12-lead ECG from an ERS case. Patient 1, whose mutation we studied in greater detail, was a 20-year-old male patient, who had experienced syncopal episodes twice while exercising. Physical examination was normal, 2D echo showed mild right atrial and right ventricular (RV) dilation with normal left ventricular and RV ejection fraction. ECG (Figure 1A) showed bradycardia [heart rate (HR) = 46 bpm] and an ERP with notching in the inferior leads, leading to a diagnosis of ERS type 2. J-point elevation ≥2 mm only can be seen in III and aVF, and an upsloping ST-segment elevation ≥1 mm was detected in V_3_-V_5_ (QTc, 367 ms; Tp-e/QT, 0.3). Patient 2 was a 17-year-old male patient presenting with spontaneous VF and episodes of syncope. His ECG (Figure 1B) exhibited sinus bradycardia (HR = 50 bpm) and an ERP with notching/slur in global leads, J point elevating prominently (≥2 mm) in leads II, III, aVF, and V_1_-V_6_, leading to a diagnosis of ERS type 3 (QTc, 363 ms; Tp-e/QT, 0.3).

**Table 2:** ECG Parameters of ERS probands with calcium mutations and healthy control.

**Index**	**Healthy control (*n* = 150)**	**ERS probands with calcium mutation (*****n*** **= 16)**			
		**Single calcium mutation (*n* = 8)**	***P-*value**	**With additional mutation(s) (*n* = 8)**	***P-*value**
HR (bpm)	72.7 ± 8.9	65.6 ± 16.1	**0.0378**	60.9 ± 9.6	** <0.001**
P wave (ms)	87.6 ± 9.1	90.9 ± 13.5	0.3318	89.4 ± 19.4	0.6133
PR interval (ms)	170.7 ± 18.7	178.0 ± 51.0	0.3448	184.3 ± 32.5	0.0567
QRS duration (ms)	89.4 ± 14.6	94.6 ± 16.1	0.3302	96.3 ± 25.9	0.2154
QTc interval (ms)	408.2 ± 21.4	386.8 ± 16.9	**0.0061**	389.5 ± 23.6	**0.0177**
Tp-e	82.3 ± 9.9	104.7 ± 18.5	** <0.001**	102.0 ± 27.6	** <0.001**
Tp-e/QT	0.22 ± 0.05	0.28 ± 0.04	**0.0011**	0.26 ± 0.07	**0.0324**

*P-value is presented in bold if P < 0.05*.

### Genetic Screening

Genetic analysis revealed 16 probands (15.4%) with variants in *CACNA1C, CACNB2*, or *CACNA2D1* genes. Eight of these (50.0%) carried additional variants in other genes, including *KCNJ8, SCN5A, SCN10A, ABCC9*, and other genes related to inherited arrhythmias. The remaining eight cases carried only one calcium-channel variant *CACNA1C*(4) and *CACNB2*(4), and none in *CACNA2D1* (Table 3). Patient 1 was identified carrying a missense mutation in *CACNA1C*. PCR-based sequencing analysis uncovered a double peak in the sequence of exon 17 of *CACNA1C* (C to T transition at nucleotide 2449), predicting substitution of proline by serine at codon 817 (P817S). In the proposed topology of the Ca_V_1.2 channel, the *CACNA1C*-P817S variant was located in a conserved site among different species in the cytoplasmic linker between domains II and III. And the mutation was indicated as “disease causing” or “damaging” by MutationTaster (0.99975), MetaLR (0.839), and FATHMMM (−3.83) with GlobalMAF 0.002.

**Table 3:** Summary of single calcium mutation in ERS probands.

	***CACNA1C***				***CACNB2b***			
Variant	P817S	G37R	G490R	E850del	S160T	A170V	S503L	R571C
Reported ID	rs112532048	rs34534613	rs121912775	rs575583988	rs149253719	NA	rs137886839	rs1060499847
Type	Missense	Missense	Missense	Frameshift	Missense	Missense	Missense	Missense
Change in nucleotide	2449C>T	109G>A	1468 G>A	2548-2550del	479G>C	509C>T	1508C>T	1711C>T
Exon location	17	2	10	19	5	6	13	13
**MAF**								
GnomAD	0.003488	0.004555	0.000162	0.00042	0.000581	NA	0.001099	NA
ExAC	0.003534	0.007385	0.000809	0.000526	0.001007	NA	0.001162	NA
1000 Genomes	0.001597	0.000399	0.000399	NA	0.001797	NA	0.001398	1.62686e-05
**SIFT**								
Score	0.212	0	0.133	NA	0.01	0.062	0.001	0
Prediction	Tolerated	Damaging	Tolerated	NA	Damaging	Tolerated	Damaging	Damaging
**MetaLR**								
Score	0.839	0.9028	0.83	NA	0.3893	0.5276	0.5305	0.6806
Prediction	Damaging	Damaging	Damaging	NA	Tolerated	Damaging	Damaging	Damaging
**MutationTaster**								
Score	0.99975	1	0.999727	NA	1	1	0.999999	1
Prediction	Disease causing	Disease causing	Disease causing	NA	Disease causing	Disease causing	Disease causing	Disease causing
**PolyPhen2**								
Score	0.999	1	1	NA	0.787	0.007	0.996	1
Prediction	Probably damaging	Probably damaging	Probably damaging	NA	Probably damaging	Benign	probably damaging	Probably damaging
**FATHMMM**								
Score	−3.83	−3.88	−3.33		−1.67	−1.81	−1.85	−2.17
Prediction	Damaging	Damaging	Damaging		Damaging	Damaging	Damaging	Damaging

### Functional Expression

**Figure 2:**
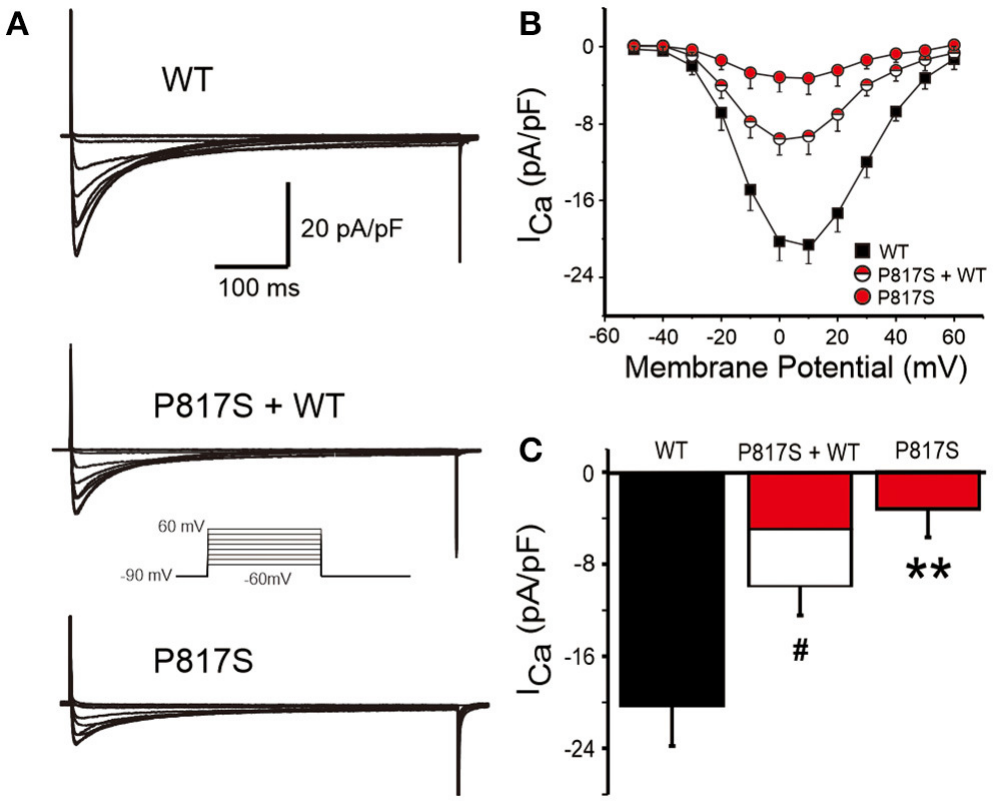
Analysis of whole-cell calcium current (*I*_Ca_) recorded from HEK293 cells expressing WT, P817S+WT, and P817S (*n* = 15, 10, 11, respectively). **(A)** Representative whole-cell *I*_Ca_ traces from WT, P817S+WT, and P817S. **(B)** Current density–voltage relationship for WT, P817S+WT, and P817S. **(C)** Bar graph showing peak *I*_Ca_ density at 0 mV from WT, P817S+WT, and P817S. ^#^*p* < 0.05, compared with P817S+WT. **p < 0.01, compared with P817S.

To explore the molecular consequences of the mutation, we cotransfected *CACNA1C*-P817S and WT with the other two subunits (*CACNB2b* and *CACNA2D1*) forming the LTCC into HEK293 cells and performed whole-cell patch-clamp experiments. Typical *I*_Ca_ tracings of voltage-dependent activation from WT, P817S+WT, and P817S mutation are shown in Figure 2A. Analysis of the current–voltage relationship (I–V curves) reveals that P817S significantly reduced the peak current density of *I*_Ca_ with 84.61% reduction at +10 mV, compared to WT (−3.17 ± 2.53 vs. −20.59 ± 3.60 pA/pF, *p* < 0.01; Figures 2B,C). Heterozygous expression of mutant also led to a 51.35% reduction.

**Figure 3:**
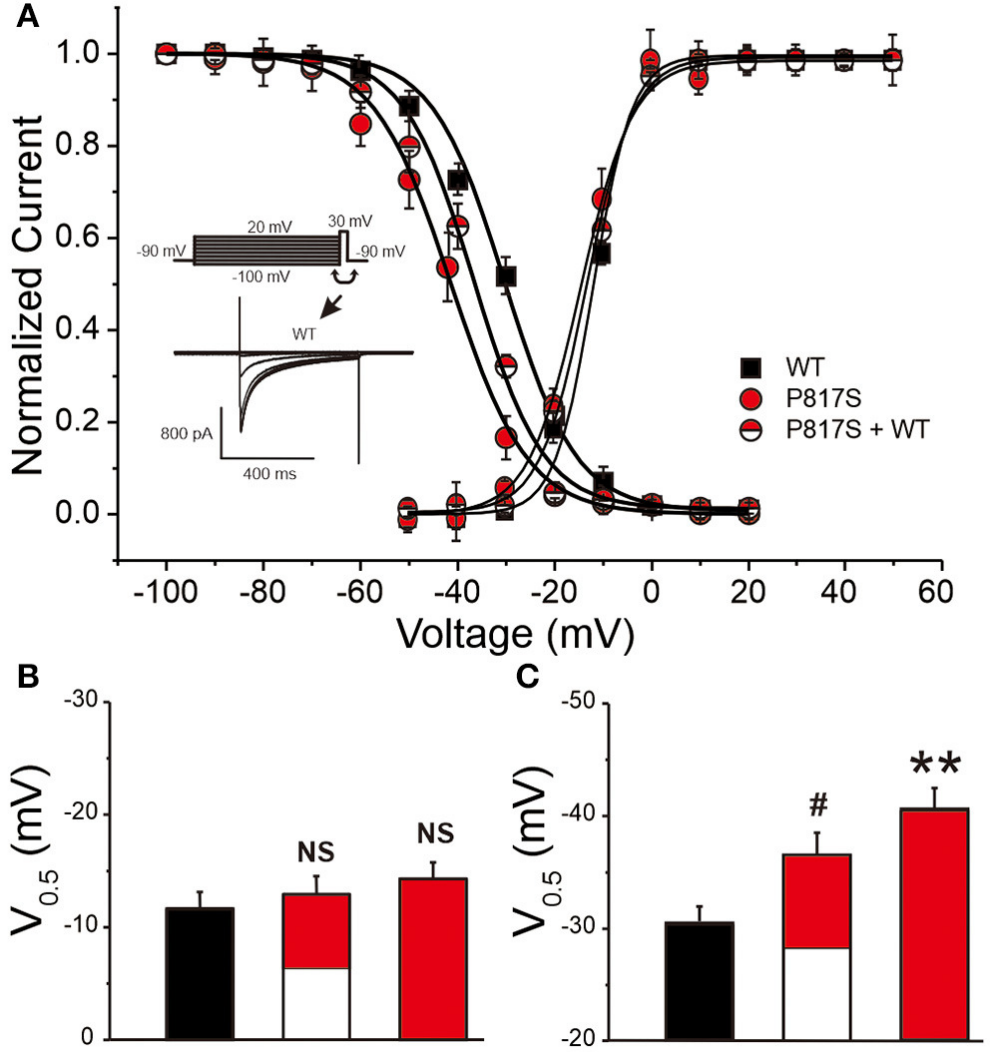
The steady-state activation and inactivation of HEK293 cells expressing WT, P817S+WT, and P817S (*n* = 15, 10, and 11 for activation; *n* = 8, 10, and 7 for inactivation, respectively). **(A)** Normalized activation conductance and inactivation current, fitted to the Boltzmann equation. **(B,C)** Bar graph showing potential for half-maximal activation and inactivation of each group. NS (no significance), #*p* < 0.05, compared with P817S+WT. **p < 0.01, compared with P817S.

The activation conductance variables (*I*/*I*_max_), obtained from the I–V curves, were further fitted with a Boltzmann function to obtain the half activation voltage *V*_½_, which displayed no significant difference among the three groups (WT vs. P817S+WT vs. P817S: −11.66 ± 0.41 vs. −13.05 ± 0.60 vs. −14.32 ± 1.24, *p* > 0.05; Figures 3A,B). Steady-state inactivation curve was also fitted by Boltzmann function, showing a significant acceleration in both P817S and P817S+WT groups (P817S vs. P817S+WT vs. WT: −40.76 ± 2.05 vs. −36.63 ± 1.98 vs. −30.53 ± 1.40, *p* < 0.01, *p* < 0.05; Figures 3A,C). The results above indicated that the *CACNA1C*-P817S mutation causes a “loss of function” (LOF) in cardiac calcium-channel activity.

**Figure 4:**
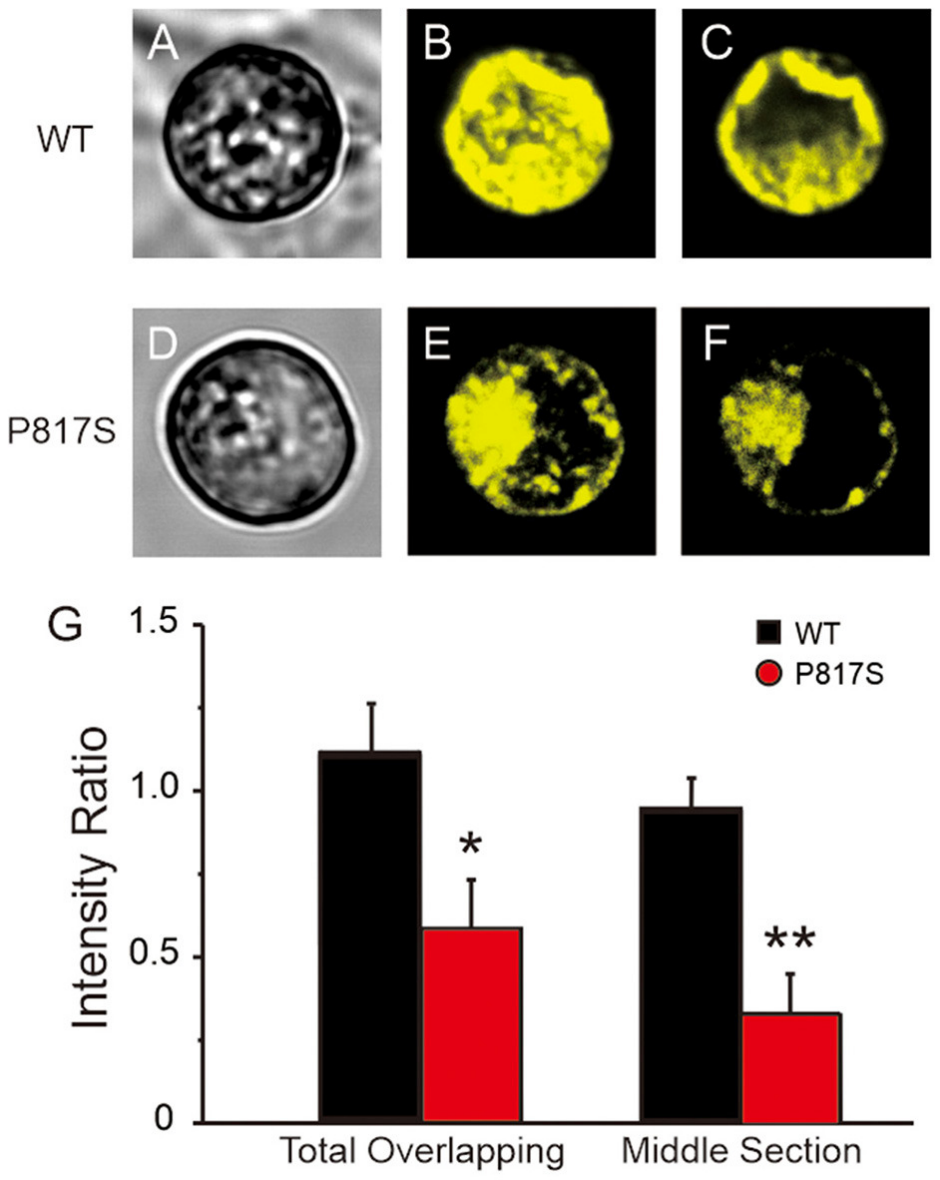
Confocal fluorescence microscopic images of EYFP-tagged Ca_V_1.2 channels in HEK293 cells expressing WT and P817S (*n* = 10 and 9, respectively). **(A–F)**, Representative confocal XYZ scans showing localization of Ca_V_1.2 channels. **(A,D)** Left, **(B,E)** middle, and **(C,F)** right photomicrographs show the light transmission images, total overlapping of phase contrast confocal images and middle section of phase contrast confocal images, respectively, for the same cell. **(G)** Bar graphs showing peripheral/central fluorescence intensity ratio of total overlapping and middle section. *p < 0.05, **p < 0.01, compared with P817S.

To evaluate whether the mutation-caused LOF was due in part to a trafficking defect, we assessed the intracellular expression pattern of WT or P817S channels tagged with EYFP by using the confocal microscopic technique. XYZ scans of WT channels on the confocal microscope showed both a central and peripheral pattern of fluorescence (Figures 4A,C), whereas the staining of P817S channels was restricted in intracellular organelles (Figures 4D,F). In the total overlapping, the ratio of peripheral/central intensity of P817S diminished (WT vs. P817S: 1.10 ± 0.16 vs. 0.58 ± 0.15, *p* < 0.05), with the decrease in the middle section more dramatic (WT vs. P817S: 0.94 ± 0.10 vs. 0.33 ± 0.12, *p* < 0.01), suggesting that P817S channels were trapped in the endoplasmic reticulum and/or Golgi complex, remaining very few localizing at the sarcolemma. These findings suggest that the loss of current observed with P817S is partially due to an impairment in trafficking of mature Ca_V_1.2 channels from the endoplasmic reticulum/Golgi complex to the cell membrane.

## Discussion

Our data show the clinical characterization and functional genetic association of ERS with cardiac calcium-channel variant, providing data in support of mutations in calcium-channel being pathogenic in ERS. In this study, a unique clinical entity in 16 unrelated ERS probands associated with genetic defects in cardiac calcium-channel is discovered. It is characterized by decreased HR and QTc, as well as increased transmural dispersion of repolarization (TDR). While an LOF in *I*_Ca_ has previously been attributed to ERS using whole-cell patch-clamp techniques, none of them further demonstrated that the impairment of membrane trafficking was the underlying mechanism of the LOF. The present study uses a confocal fluorescence microscope to verify that impaired membrane trafficking of calcium-channel contributes to the LOF in *I*_Ca_ caused by *CACNA1C*-P817S (Figure 4). This is the largest one for studying calcium-related ERS by far.

ERS shares many similarities in terms of clinical perspective with BrS, such as male predominance, the average age of first arrhythmic events, and condition of arrhythmic episodes, suggesting similar pathophysiology; thus, these two entities are referred to as J-wave syndromes (JWS) (2). In genetics, multiple ion-channel mutations have been linked with ERS. The first gene mutation locates in *KCNJ8* (12, 13), causing a gain-of-function of I_K−ATP_; others include LOF mutations in *CACNA1C, CACNB2, CACNA2D* (17), *SCN5A* (15), and *SCN10A* (14) and gain-of-function mutations in *ABCC9* (14) and *KCND3* (16).

*CACNA1C* lies in chromosome 12, coding for the main pore-forming α1 (Ca_V_1.2) subunit of the cardiac LTCC. The genetic defects of *CACNA1C* can precipitate many cardiac syndromes, such as Timothy syndrome (TS) (22), long QT syndrome (LQTS) (23), BrS, and ERS (18). The discovery of *CACNA1C-*encoded LTCC mutation in JWSs manifested that LOF perturbations in cardiac LTCC could have drastic phenotypic implications, such as male predominance, accentuated J waves, and ST-segment elevation when bradycardia or pauses happen, relative shortened QTc interval, unexplained syncope, and SCD (18). Since then, studies identify a *CACNA1C* mutation (E850del) that cosegregates with ERS phenotype in a pedigree with ERS-associated SCD and confers a markedly decrease in peak *I*_Ca_ density (17, 24). Since then, Chen et al. and Liu et al. confirm that the LOF of *I*_Ca_ caused by *CACNA1C*-R1973P or *CACNA1C*-Q1916R can induce ERS (25, 26). Understanding the role of cardiac calcium-channel mutation in the etiology of ERS is limited.

The initial event of ERP may be cardiac arrest, which is often the presenting episodes of VF, and male predominates among those patients with cardiac arrest related to ERP (>70%) (5). Nevertheless, VF is rare, whereas ERP is a relatively common electrocardiographic phenomenon; it might not happen until the third decade of life, perhaps due to relatively higher testosterone levels in this stage (5, 27, 28). A history of syncope at rest has a strong association with ERS, which is due to pause-dependent augmentation of J waves and ST-segment elevation ahead of VF, so does bradycardia (27). Report discovers ERP has evidence for a heritable basis in the general population, or ERP owns a heritability (10). Family history of SCD is important information we should pay attention to in clinical practice. However, only 12.5% of ERS probands are found to have a familial history of SCD in our study, not far from what Haïssaguerre et al. found (16%) (5). Nevertheless, if a positive family history of unexplained SCD at a young age is discovered, systematic evaluations of all surviving family members are necessary regardless of the presence or absence of ERP. Besides, we found three patients (18.8%) present with AF, a higher prevalence than in the general population. Watanabe et al. reported that AF is found in 23% of their cases with ERS, and AF has been reported in 15% of ERS patients in the investigation of Hwang et al. and 22% in the research of Kamakura et al., implying high overlapping between AF and ERS (29–31). A report also demonstrates that ERP is more common in patients younger than 60 years with lone AF than healthy controls and declares that ERP may indicate susceptibility to AF (32). Causative genes of ERS encode ion channels including sodium, calcium, and potassium channels. Given the affected protein is present in ventricular and atrial myocardium, the repolarization abnormalities of the atrial and ventricular may be potentially related. Interestingly, all of the causative genes of ERS have also been associated with AF. There may be common genetic background for AF and VF related to ERP (32).

TDR has been proposed to underlie arrhythmogenesis in JWS, including ERS and BrS (33). Electrocardiographic markers reflecting TDR include Tp-e interval and Tp-e/QT ratio and increased Tp-e interval and Tp-e/QT ratio have been associated with IVF development in ERS and therefore may be considered as non-invasive markers of arrhythmogenesis (34–36). In the process of looking for JWS candidate genes, Antzelevitch et al. found three (60%) of five BrS probands who present with a short QT interval carried a calcium-channel mutation, which points to genetic heterogeneity for this phenotype, being the first to propose LTCC genes as cause for BrS associated with short QT. A decrease in *I*_Na_ or *I*_Ca_ or augmentation of any one of outward currents, including *I*_Kr_, *I*_Ks_, and *I*_to_, can cause a net repolarizing current, resulting in an accentuated action potential dome, manifesting as the augmentation of the J wave or appearance of ST-segment elevation in ECG. A further increase in net repolarizing current can result in partial or complete loss of the action potential dome, leading to a transmural voltage gradient that manifests as greater ST-segment elevation. *I*_Ca, L_ is the main current of phase 1 of the action potential; a decrease of *I*_Ca, L_ may shorten the action potential duration (37, 38). In our data, the average QTc is 386.8 ± 16.9 ms among those who carry calcium mutation alone, which falls into the shorter side of the range of QTc interval. As to the half with additional variation(s), 62.5% carry variations in previously reported QT-prolonging genes, including *SCN5A* (39) and *SCN10A* (40). Slower HR, shorter QTc, and longer Tp-e/QT are also found in those compound mutations carriers. That implies the unique clinical phenotype or entity aroused by cardiac calcium mutation is dominant in ERS cases.

The majority of ERP subjects will remain asymptomatic in practice with a relatively low prevalence of arrhythmic events or SCD. Rosso et al. indicate that the presence of J wave on the ECG increases the probability of VF from 3.4:100,000 to 11:100,000 (27). Thus, careful attention should be devoted to risk assessment for the development of life-threatening arrhythmias in potential ERS cases. Risk stratifications may encompass but are not limited to the following: high-amplitude J waves (≥0.2 mV), J waves with horizontal or downsloping ST segment, documented VF or documented polymorphic VT, dynamic changes in J-wave amplitude, positive family history of SCD or arrhythmic syncope, prolonged Tp-e interval, identified gene mutations, and so on. First, either slurred or notched J-point elevation ≥0.2 mV appears to be associated with an increased risk, although relatively rare in the general population (9). And horizontal or descending ST segment following J-point elevation is associated with a worse prognosis than an ascending ST segment (41). Furthermore, J-point elevation in IVF is of greater amplitude and wider ECG lead distribution than those with an established cause of cardiac arrest (8). The family history of SCD in subjects with ERP has been proven to be another risk factor (42). The study also suggests that an increased Tp-e/QT ratio, which reflects TDR in subjects with ERP, may be a specific ECG marker for increased arrhythmic risk (43). In the present study, we found 10 (62.5%) suffered IVF, 4 of them (40.0%) presented with a marked J-point elevation (≥2 mm), and 3 (30.0%) showed a global pattern (ERS3). Our patient 2 displays all these traits and presents with severe clinical manifestation, which calls for a close follow-up. These patients are at higher risk of life-threatening arrhythmias, and more active therapies should be administered.

Further genetic analysis detects a missense mutation, P817S-*CACNA1C*, in patient 1, pointing to genetic heterogeneity for this phenotype. Heterologous expressions and patch clamping techniques are conducted to illustrate the disease-causing ability of the mutation; we found out that the P817S mutant dramatically decreases peak current density by 84.61%, and the steady-state inactivation is also significantly accelerated, displaying an LOF of *I*_Ca_. Further confocal microscopy study reveals a decreased trafficking of Ca_V_1.2 protein caused by P817S mutation. Decreased *I*_Ca_,_L_ leads to augmented net outward currents and shortens the cardiomyocyte repolarization period. Because of the transmural discrepancies of outward potassium currents (*I*_to_, *I*_K_, etc.), the enhancement in net outward current results in partial or complete loss of the action potential dome, leading to a transmural voltage gradient that manifests as J waves. Otherwise, accelerated repolarization caused by the mutation could lead to a pursuant short QT interval in the surface ECG. Boczek et al. revealed that *CACNA1C-*P857A induces LQTS by causing a gain-of-function of *I*_Ca, L_ and an increase of Ca_V_1.2 membrane trafficking and speculates the modulation might be because the mutation locates at the conserved proline, glutamic acid, serine, and threonine rich (PEST) domain of Ca_V_1.2 II-III loop, which acts to proteolytic signaling through the cellular “quality control” system (PEST1 is S446/459, and PEST3 is S840/861) (23). However, as early as 2007, *CACNA1C-*A39V inducing LOF due to a trafficking defect in BrS is found by Antzelevitch et al. (18). Yet, it still remains unclear why *CACNA1C* mutations can cause the trafficking decrease/defect of the channels to the plasma membrane. Trafficking defects are considered to involve misfolding or improper assembly of the protein structure, leading to its retention in the endoplasmic reticulum and degeneration without transport to the Golgi complex by the “quality control” system, in which lectin-like endoplasmic reticulum chaperones play a key role. Substitution of proline with serine has significant meanings for protein folding and thereby causes trafficking abnormalities and degradation. Tertiary post-translational processing may be affected and therefore influences trafficking to the cell membrane.

### Study Limitations

One limitation of our study may be the small number of affected individuals, which precludes us from reaching a more definitive conclusion, although this cohort has already been the largest one for studying calcium-related ERS by far. Another one is the mechanism of trafficking decrease caused by *CACNA1C*-P817S. To observe the progressive nature of the disease, we plan to further follow up with these patients, especially those with a high risk of life-threatening arrhythmias. The work led by our coauthors has demonstrated the first human induced pluripotent stem cell (hiPSC) model of ERS recently (44). hiPSCs from the ERS probands with calcium-related mutation are also projected in our schedule, to replicate the phenotype and perform further research on stem cell level.

## Conclusions

The present study demonstrates that ERS caused by cardiac calcium-channel variant presents unique clinical features, including decreased HR and QTc, as well as increased transmural heterogeneity. Further functional investigations provide solid evidence that *CACNA1C*-P817S is a mutation causing impaired membrane trafficking of Ca_V_1.2 protein, inducing an LOF of *I*_Ca_, thus leading to the manifestation of ERS phenotype.

## Data Availability Statement

The datasets generated for this study can be found in online repositories. The names of the repository/repositories and accession number(s) can be found in the article/Supplementary Material.

## Ethics Statement

The studies involving human participants were reviewed and approved by Renmin Hospital of Wuhan University Institutional Review Board and performed in accordance with the declaration of Helsinki. Written informed consent to participate in this study was provided by the participants or their legal guardian/next of kin.

## Author Contributions

HB-M and DH designed the study performed clinical phenotyping of study subjects, supervised, and coordinated the genetic laboratory work. XC, HB-M, HX, BY, HJ, CA, and DH coordinated the clinical evaluations. XC, HB-M, ZZ, and DH performed electrophysiology and confocal study. XC, HB-M, ZZ, GC, and DH organized and summarized the database. XC, HB-M, and DH analyzed the data. GC, BY, HJ, and DH developed the conceptual approaches to data analysis. XC, HB-M, CA, and DH wrote the manuscript. All authors contributed to editing the manuscript.

## Conflict of Interest

CA serves as a consultant and received grant funds from Novartis and Trevena Inc. The remaining authors declare that the research was conducted in the absence of any commercial or financial relationships that could be construed as a potential conflict of interest.
